# Building lipids for myelin

**DOI:** 10.18632/aging.101458

**Published:** 2018-05-22

**Authors:** Laura Montani, Ueli Suter

**Affiliations:** 1 Institute of Molecular Health Sciences, Swiss Federal Institute of Technology, ETH Zurich, Switzerland

**Keywords:** nervous system, myelin, glia, Schwann cells, lipids, fatty acids, fatty acid synthase, FASN

Various lipids are essential constituents of diverse biological membranes. With the exception of cholesterol, such membrane lipids share the use of fatty acids as building blocks. Besides structural functions, fatty acids and the derived lipids are also involved in other central biological processes, such as providing an important energy source, acting as signaling molecules, being modulators of gene regulation, and generating post-translational modifications of proteins [1]. However, the contributions of fatty acid metabolism to healthy development, in homeostasis, aging, diseases, and after injury, remain to be fully explored. In particular, for many cell types we are still lacking a detailed understanding of the dependence and dynamics of cell-endogenous *de novo* fatty acid synthesis versus uptake both from the tissue environment and the circulation, together with the precise knowledge of the underlying regulatory mechanisms under different physiological conditions. Myelinating glia of the nervous system are in extraordinary high demand for lipids which are required to form the multi-layered membranous myelin sheaths that enwrap and insulate axons, allowing and refining efficient neuronal impulse propagation. This reliance is highlighted by various myelin pathologies that are observed in several diseases affecting lipid metabolism [2]. In particular, cholesterol synthesis is crucial for correct myelination [3]. This can be explained by the fact that selectively permeable barriers to the circulation shield the nervous system from circulating cholesterol later in development, so that endogenous synthesis becomes obligatory. In contrast, fatty acids can, at least partially, be transported across these barriers allowing especially the crucial uptake of essential fatty acids [4]. Nevertheless, cells also have the ability to produce non-essential fatty acids which is critical under increased metabolic demand such as in highly proliferating cells [5]. This process requires fatty acid synthase (FASN), the enzyme that guides the production of palmitic acid which is then used as substrate to synthesize more complex fatty acids.

We found recently that FASN plays an essential role in Schwann cells, the myelinating glia of the peripheral nervous system [6]. Lack of FASN-mediated *de novo* lipid synthesis in Schwann cells of genetically modified mice caused a partial block in the onset of myelination. Further analyses revealed that lipogenic activation of the transcriptional network regulated by the transcription factor PPARγ contributes to driving the initiation of myelination [6]. However, due to the high diversity of lipid-mediated functions, we anticipate that future studies will also uncover other mechanisms contributing to the fatty acid-mediated regulation of myelination.

In our experimental setting, some Schwann cells still managed to start myelination in the absence of FASN. Why would this occur? Our findings suggest that one answer may lie in the relative localization of these cells in the tissue, more specifically in their proximity to blood vessels as a source for fatty acid uptake. Whether these observations reflect a regulated compensatory mechanism remains to be clarified. In addition, we found that adipocytes associated with peripheral nerves responded to a lack of fatty acid synthesis in Schwann cells by undergoing lipolysis suggesting another potential compensatory reaction [6]. We speculate that such mechanisms might also be involved in the regulation of particular physiological states in health, after injury, and/or in disease.

Remarkably, increasing dietary intake of fatty acids by putting the mutant mice on a high-fat diet did not ameliorate the observed impaired myelination. Instead, this treatment resulted in worsening of the phenotype in nerves of mutant mice, although no effect was observed in nerves of control mice [6]. Further investigations aimed at understanding how diets modulate the lipid state of the nervous system are required to follow up on these studies. Additionally, the specific mechanisms employed by diverse nerve cells to take up, store, and metabolize fatty acids, with which physiological consequences, remain to be explored further.

Many questions remain open in the context of our study. Most closely related, the detailed roles of *de novo* fatty acid synthesis and fatty acid-based regulation in establishing and refining suitable connectivity in the central nervous system remain a major task for the future [7].
